# Obituary

**Published:** 1869-08-15

**Authors:** 


### *Extracts from the Medical Press.*

We copy notices, from several reliable sources, of E. Fougera's preparation of Cod Liver Oil, believing it worthy of notice; also call attention to his advertisement in our columns:

*Medical Gazette of New York City, March* 21, 1868.

"The advantages claimed for Mr. E. Fougera's Cod Liver Oil, are that by reason of the addition of Iodine, Bromine, and Phosphorus, it is more efficacious, and at the same time the stomach need not be disordered by an excessive amount of oil administered. This oil was given to about eighty patients in the out-door department of Bellevue Hospital, about thirty of whom were children, the remainder belonging chiefly to the department of chest diseases. The opinion of the physicians using it are nearly unanimous to this effect: That the oil is of a decided medicinal value; that, compared with ordinary cod liver oil, it appears to take effect more rapidly; and that it obviates the very common necessity of adding extemporaneously to the oil, medicines containing iodine or iron, particularly to the syrup of the iodide of iron." L. M. Yale, M. D., Editor.

*St. Louis Medical Reporter, April* 1, 1867, *St. Louis, Mo.*

"We have, personally, used Mr. Fougera's Compound Iodinized Cod Liver Oil, and can, from experience, pronounce it one of the best articles of the kind now in use, and trust it will receive that attention from the profession which it so deservedly merits. His other preparations stand high, both for excellence and purity.

J. S. B. Alleyne, M. D., C. F. Potter, M. D., Editors.

